# A Novel, Highly Selective RT-QPCR Method for Quantification of MSRV Using PNA Clamping Syncytin-1 (ERVWE1)

**DOI:** 10.1007/s12033-015-9873-2

**Published:** 2015-05-15

**Authors:** Grzegorz Machnik, Estera Skudrzyk, Łukasz Bułdak, Krzysztof Łabuzek, Jarosław Ruczyński, Magdalena Alenowicz, Piotr Rekowski, Piotr Jan Nowak, Bogusław Okopień

**Affiliations:** Department of Pharmacology, Medical University of Silesia, Medyków 18, 40-752 Katowice, Poland; Faculty of Chemistry, University of Gdańsk, Wita Stwosza 63, 80-308 Gdańsk, Poland; Department of Nephrology, Hypertension, and Kidney Transplantation, Medical University of Łódź, Pomorska 251, 92-213 Łódź, Poland

**Keywords:** Peptide nucleic acids (PNAs), Endogenous retroviruses, Human, Syncytin-1 glycoprotein, Mutation detection, Multiple sclerosis-associated retrovirus (MSRV)

## Abstract

HERV-W is a multi-locus family of human endogenous retroviruses (HERVs) that has been found to play an important role in human physiology and pathology. Two particular members of HERV-W family are of special interests: ERVWE1 (coding syncytin-1, which is a glycoprotein essential in the formation of the placenta) and MSRV (multiple sclerosis-associated retrovirus that is thought to play a significant role in human pathology as a result of its increased expression in the brain tissue and blood cells derived from patients with multiple sclerosis (MS)). Both ERVWE1 and MSRV mRNA share high level of similarity and hence a method that allows to exclusively quantify the MSRV expression in clinical samples would be desirable. We developed a quantitative polymerase chain reaction (QPCR) technique for the detection and quantification of the multiple sclerosis-associated retrovirus. The assay utilises fluorescently labelled oligonucleotide probe, which is complementary to the conservative fragment of MSRV *env* gene and a peptide nucleic acid (PNA) probe, fully complementary to the ERVWE1 sequence fragment that efficiently blocks the polymerase action on ERVWE1 templates. The PNA molecule, if used parallel with hydrolysis probe in QPCR analysis, greatly facilitates the detection efficiency of MSRV even if ERVWE1 is present abundantly in respect to MSRV in the analysed sample. We achieved a wide and measurable range from 1 × 10 e^2^ to 1 × 10 e^8^ copies/reaction; the linearity of the technique was maintained even at the low MSRV level of 1 % in respect to ERVWE1. Using our newly developed method we confirmed that the expression of MSRV takes place in normal human astrocytes and in human umbilical vein endothelial cells in vitro. We also found that the stimulation of human monocytes did not influence the specific expression of MSRV but it caused changes in mRNA level of distinct HERV-W templates.

## Introduction

Human endogenous retroviruses of the HERV-W family are broadly dispersed thorough the human genome; it is estimated that HERV-W are represented by more than one hundred copies per haploid genome [1]. According to Pavlicek et al. [2] and their in silico evaluation, this retroviral group comprises 77 proviral copies with complete or, at least, partial internal (non-LTR) sequences, and of 343 long terminal repeats without any internal sequences (solo-LTRs). Despite the huge number of HERV copies, only a few intact open reading frames (ORFs) survived, avoiding the gradual accumulation of compromising mutations and/or deletions. The role of retroviral sequences in human genome has been broadly investigated recently, as there is numerous evidence of their adverse effect upon (auto-) immunologic disorders. Some gene expression products of human endogenous retroviruses have been detected as transcripts and as proteins in the central nervous system and they have been frequently connected with neuroinflammation. The HERV-W family has received substantial attention in large part because of its associations with diverse syndromes including multiple sclerosis (MS) and several psychiatric disorders, such as schizophrenia [3–5]. It has been shown that both HERV-W and HERV-H/F were specifically activated in the circulation and in the central nervous system (CNS) in the majority of MS patients and, particularly, the envelopes (*env* mRNA and *env* proteins) appear to be strongly associated with disease activity and progression [6]. Brudek et al. found a significantly higher expression of HERV-H and HERV-W *env* epitopes on B cells and monocytes from patients with active MS compared with patients with stable MS or control individuals [7]. Another known member of the HERV-W family is ERVWE1, which is located on chromosome 7, coding syncytin-1. This glycoprotein has highly fusogenic properties that are essential during the development of syncytiotrophoblast in placenta [8]. Both multiple sclerosis-associated retrovirus (MRSV) and ERVWE1 mRNA are very similar to each other. MSRV and ERVWE1 *env* sequences belonging to the HERV-W family are distinct from other HERV families, showing only 38–47 % sequence similarity with HERV-FRD/syncytin-2 or HERV-H and HERV-K and also differ from exogenous human retroviruses, such as human T-lymphotropic virus 1 and human immunodeficiency virus (20–25 % similarity) [9]. On the other hand MSRV and ERVWE1 pol region share 92 % of sequence while their *env* genes are identical in 81 % [10, 11].

Because an increased level of HERV-W RNA has been reported in brain tissue from MS patients [12], a precise technique that allows differentiating between MSRV and syncytin-1 (from ERVWE1 locus) mRNA would be very desirable. Indeed, an increased level of ERVWE1, but not MSRV mRNA in MS brain samples has been reported [11]. The most popular method that was also utilised by researchers from Prof. Power’s group [13], is to use allele-specific oligonucleotides (ASO) as primers in the amplification reaction of HERV-W template. This technique allows the selective amplification of mutant or wild-type gene fragment and the principle of the method is based on the phenomenon that DNA polymerase cannot bind to the 3′end of the primer if there is a mismatch between oligonucleotide and the template [14]. However, as shown by Kwok et al., many 3′ mismatches between a primer and its template do not significantly impair the PCR reaction [15]. Moreover, Yoshida et al. noted that if they used hydrolysis probes in real-time PCR settings, it was difficult to measure the amounts of hepatitis-B virus mutants accurately, especially when the target strain was only a minor component of the mixed population [16]. Some analogy is observed in the case of HERV-W family where the target i.e. MSRV mRNA level may comprise only a small portion of abundantly expressed ERVWE1 mRNA.

Peptide nucleic acid (PNA) is a DNA mimicking synthetic oligonucleotide with peptide back-bones instead of sugar groups and was first reported in 1991 by Nielsen et al. from the University of Copenhagen [17]. By comparison with nucleic acid, PNA is much more stable chemically and biologically and, what is particularly noteworthy is, it is resistant against nucleases [18]. It has been documented that PNA sequence (probe) recognises and binds to the complementary DNA template with higher affinity and specificity than DNA-to-DNA does, because of the lack of electrostatic repulsion. On the other hand, high affinity and binding strength of PNA/DNA duplexes dropped drastically if even a single nucleotide mismatch occurs in the target DNA sequence. This phenomenon presented the opportunity to utilise PNA oligomers in single nucleotide polymorphism (SNP) analysis [17]. Peptide nucleic acid cannot be extended by the DNA polymerase, therefore it cannot serve as a primer in polymerase chain reaction (PCR) as well. It can, instead, be useful as a PCR blocking (clamping) molecule that effectively prevents primer hybridisation and subsequent amplification of undesired (wild type) template. If standard oligonucleotide PCR primers are designed to overlap the SNP site in the target (mutated) sequence and a PNA oligomer that is fully complementary to the other (wild type) DNA is added into the reaction, PNA preferentially hybridises (binds) to the wild type but not to the mutated site DNA. This, in turn, strongly reduces the amplification of the wild type relative to the mutant DNA. In such a setting, only the amplification of the sequence of interest is allowed.

As mentioned above, PNA-mediated PCR clamping method is especially desired if DNA of interest is present in a very low amount relative to the wild type one [19]. This situation can occur, for example, when only a few mutant cancer cells have to be detected along with a large excess of healthy cells in a tissue biopsy or for the seeking of a drug-resistant mutant among heterogeneous viral population [20]. As shown by Kim et al., this method allows SNP detection even when the wild-type DNA is present in up to 20,000-fold excess relative to the mutant [19]. This unique feature characterising the clamping PCR assay is especially important in the analysis of HERV-W family because ERVWE1, being physiologically active in many cells, expresses abundantly and may overshadow possible discrete differences of MSRV level in investigated samples.

In our previous report, we described a real-Time polymerase chain reaction/PNA-mediated methodology that allows the specific quantitative amplification of multiple sclerosis- associated retrovirus (MSRV). The assay is based on two sequence-specific oligonucleotides (PCR primers) that are common for both MSRV and ERVWE1 gene fragment, an ERVWE1- specific PNA probe and SYBR Green 1 intercalating dye that was essential in the quantitative assessment. Those results showed that it is possible to estimate the MSRV level in a biological sample if the ERVWE1 template is excluded from the reaction by means of PNA-mediated PCR clamping. According to our results with the PNA inhibition of ERVWE1, we showed that the overall expression of HERV-W was similar in samples treated with PNA or without PNA clamping, therefore we concluded that the expression of MSRV is predominant over syncytin-1 in human astrocytic U-87 MG cell line. Because the calculated HERV-W *env* mRNA copy number differed in respect of the presence or absence of the ERVWE1-specific PNA, therefore we concluded that MSRV is present predominantly over syncytin-1 in human astrocytic U-87 MG cell line. Additionally, compared to the intercalating dyes (e.g. SYBR Green I), our novel method incorporated the specific fluorescent probe that is much more suitable for virology/microbiology experiments, due to higher pathogen-specificity of the procedure primers, the utilisation of a third sequence-specific element, such as oligonucleotide probe is preferred over an intercalating dye in the virology/microbiology fields due to higher pathogen-specificity of the method. In the probe-based chemistry, both primers and probe hybridise to the common target, thus as much as three events must occur independently to give a specific fluorescent signal [21].

## Materials and Methods

### In Silico Analysis of HERV-W Sequences

HERV-W *env* sequences were derived from the GenBank database according to previously published data [10, 11]. We compared three *env* sequences denoted as MSRV with four *env* sequences denoted as coding for syncytin-1. Accession numbers of the investigated entries were as follows: AF331500, AF127229, AF072498 for MSRV and AY101582, AF513360, AF208161 and NM_014590 for syncytin-1. In both sequence types, a consensus sequence was built using CONS software from the EMBOSS package [22]. Whole design was then focused only on a highly conserved common fragment for all MSRV sequences and for syncytin-1 sequences, which was located between nucleotide position 1187 and 1456 in respect to the reference sequence (NM_014590). We paid attention on this region as it bears several point mutations that are specific and conservative for MSRV or syncytin-1 sequences only and therefore provides an appropriate place for the hybridisation of sequence-specific PNA and hydrolysis probe.

Peptide nucleic acids, 15-nucleotides in length, were synthesised and purified at the Department of Chemistry, University of Gdańsk, Poland (please see “Synthesis of peptide nucleic acids complementary to ERVWE1” caption for details). Lyophilised PNA was resolved in 5 % DMSO and left for 20 min at room temperature (RT) to obtain a stock concentration of 10 μM. PNA was then aliquoted and stored at −20 °C.

**Fig. 1:**
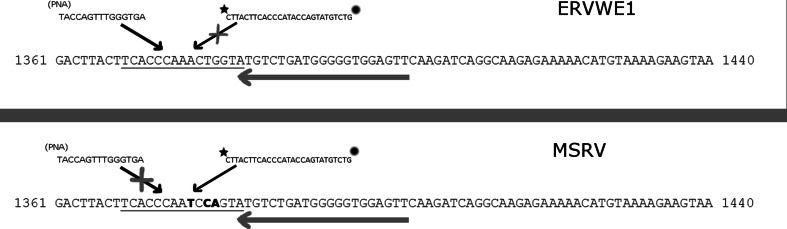
A principle of the method behind the quantitation of MSRV expression using PNA-mediated quantitative polymerase chain reaction. Peptide nucleic acid (PNA) probe (on the *left-hand side* of the figure) binds to the ERVWE1 template with high affinity due to 100 % complementarity of their sequences and prevents the hybridisation of fluorescently labelled probe (on the *right*). The probe remains intact and therefore no signal is emitted. If MSRV serves as template, PNA probe cannot bind tightly to the DNA due to three mismatches between PNA and template that diminish drastically its affinity. On the contrary, fluorescent probe now is allowed to bind to the MSRV template and afterwards it is cleaved by the polymerase during the elongation phase and emits a signal as normally seen in probe-based QPCR

Oligonucleotide primers for polymerase chain reaction were designed according to previously published guidelines [23]. Three reverse and one common forward primer were chosen for further analysis. Reverse primers were located in different positions in respect to the target region for PNA and for the probe. The reciprocal primers, PNA and probe hybridisation regions in MSRV and ERVWE1 *env* gene sequence are shown in Fig. 1. All oligonucleotides that were used in our work are also specified in details in Table 1.

**Table 1:** Oligonucleotide primers used in the work—a summary

Oligonucleotide name	Sequence	Remarks
Syncytin_out_F	5′-TGCCCCATCGTATAGGAGTC-3′	For the cloning procedure, PNA-mediated PCR and megaprimer mutagenesis
Syncytin_out_R	5′-CGGGTGAGTTGGGAGATTAC-3′	For the cloning procedure
sync1_REV/-2^*)^	5′-ATTCCACCCCCATCAGACATA-3′	^*)^ Reverse primers used in the PNA-mediated PCR clamping together with Syncytin_out_F as forward primer
sync1_REV/-1^*)^	5′-AATTCCACCCCCATCAGACAT-3′	As above
sync1_REV/0^*)^	5′-GAATTCCACCCCCATCAGACA-3′	As above
Mutagenic MRSV	5′-CAGACATACTGGTATGGGTGAAGT-3′	For use in the megaprimer mutagenesis technique
PNA ERVWE1	N′-TACCAGTTTGGGTG-C′	For the PNA-mediated PCR clamping of ERVWE1
PNA ERVWE1-T	N’-TACCAGTTTGGGTGA-C’	For the PNA-mediated PCR clamping of ERVWE1
MSRV Probe	FAM 5′-CTTACTTCACCCATACCAGTATGTCTGATG- 3′ BHQ1	For the quantitation of MSRV sequences by means of QPCR

### Extraction of Ribonucleic Acids and Reverse Transcription Reaction

Total RNA was extracted from normal human astrocytes (NHA) cell line (Lonza Ltd, Warsaw, Poland), human umbilical vein endothelial cells (HUVECs) (Sigma-Aldrich, Poznań, Poland and from human monocytes/macrophages. The study was accepted by the Bioethical Committee of the Medical University of Silesia in Katowice, Poland. The investigation conformed to the principles of the Declaration of Helsinki. Human whole-blood samples were taken from antecubital veins of healthy volunteers (aged 18–40 years old). Peripheral blood monocytic cells (PBMCs) were obtained after centrifugation on blood in the Histopaque (Sigma-Aldrich Co., Poznań, Poland). Afterwards monocytes were isolated from the PBMCs by negative immunomagnetic separation using Pan-T and Pan-B Dynabeads (Dynal, Oslo, Norway) [24]. Monocyte-derived macrophages were then stimulated with medium supplemented with 1 μg/ml of lipopolysaccharide (LPS, Escherichia coli serotype 0111:B4, Sigma, Germany) as described previously [25]. Control cells were maintained at the same conditions except the addition of LPS-containing medium.

For NHA and HUVECs, 3 ml of TRI reagent (MRC Inc., USA) was used to lyse cells from one 10 mm in diameter tissue culture plate after the >80 %-confluence was achieved. Before TRI reagent was added, culture medium was removed but without washing the cells with PBS. Monocyte cultures were lysed using one ml of TRI reagent, which was added directly into wells of a 24-well culture plate after removing the culture medium. Three wells were then pooled together. After cell lysis and complete disruption of nucleoprotein complexes, total RNA was precipitated and further purified according to the one-step extraction method described by Chomczynski et al. [26]. Finally, ribonucleic acids were resolved in 50 μl of deionised formamide (Formazol, MRC Inc., Cincinnati, Ohio, USA). RNA samples were examined and quantified spectrophotometrically at wavelengths of 260 and 280 nm. One μg of total RNA was diluted in nuclease-free water and treated with 1.2 U of Recombinant DNase I, RNase-free (Ambion-Life Technologies Corp., Carlsbad, California, USA) in order to remove any residual DNA present in RNA extracts. This step was crucial for further interpretation of results as HERV-W amplicons of both cDNA and genomic DNA origins are of the same length, and it would not be possible to utilise intron/exon spanning primers. After DNase treatment we performed additional quantitative PCR assays of RNA and DNA samples without reverse transcriptase (RT negative), to exclude the possibility of RNA contamination by DNA. After the QPCR, the reaction mixtures were loaded onto agarose gel. Neither the QPCR plots nor theagarose gel analysis gave positive signals for that samples. In the next step, 500 ng of DNA-free RNA was reverse-transcribed using GoScript Reverse Transcription System (Promega Corp. USA) in the final volume of 20 μl. Reverse transcription (RT) reaction contained of 1×Reverse Transcription buffer, 4.0 μl of 25 mM MgCl_2_, 2.0 μl of 10 mM dNTP mixture, 0.5 μl of RNasin Ribonuclease Inhibitor, 15 units of AMV Reverse Transcriptase and 0.5 μg of Random hexamers. Reaction mixture was incubated for 10 min at room temperature and then for 30 min at 42 °C followed by thermal inactivation of reverse transcriptase at 95 °C for 5 min. According to the manufacturer’s recommendations, reverse transcription reaction mixture was further diluted 1:4 with nuclease-free water before the use in QPCR. Five μl of diluted RT reaction mixture (i.e. an equivalent of about 25 ng of total RNA) was then used as a template for subsequent QPCR analysis.

### The Choice of a Proper Priming in Reverse Transcription Reaction

In our assay, we used random hexamers as primers for the reverse transcriptase enzyme. As it is stated in Promega’s instruction for use of their product # A3500 “Choose Oligo(dT)15 when priming at the 3′poly(A) region is desired. Choose Random Primers when priming throughout the length of the RNA is desired”.

It should be emphasised that the choice of optimal RT priming is crucial to get the most representative cDNA pool. Human endogenous retroviral-derived particles make even more complex issue because they are broadly dispersed thorough the genome and are mostly truncated; e.g. a significant part of HERV-W possesses a poly-A tail (such as syncytin-1) while other retroviral members does not. If so, a mixture of oligo(dT)_15_ and random primers may, in theory, improve the quality of cDNA pool. However, the use of oligo(dT)_15_ concomitantly with random hexamers/nonamers is problematic because both solutions require slightly different thermal conditions and finally the resulting cDNA library may not be more representative than when using random primers alone.

For cloning purposes, DNA extract obtained from human placenta was used (a kind gift from Prof. dr Urszula Mazurek, Dept. of Molecular Biology, Medical University of Silesia, Sosnowiec, Poland).

### The Amplification of Syncytin-1 (ERVWE1) Gene Fragment and Site-Directed Mutagenesis

Polymerase chain reaction was performed using Qiagen Taq PCR Master Mix kit (Qiagen, Germany). The reaction mixture in a total volume of 25 μl consisted of 12.5 μl of 2× Qiagen PCR Master Mix, 200 nM of each Syncytin_out_F and Syncytin_out_R primer and 0.5 μl of placental DNA extract as a template. After reaction, amplicons were resolved by means of agarose gel electrophoresis, excised from the gel and extracted/purified using silica-based spin columns (Invisorb Fragment CleanUp, InviTec, Germany). Four separately purified amplimers were then sequenced (Genomed SA, Warsaw, Poland). BLAST analysis of obtained sequences confirmed that the specific amplification of ERVWE1 locus took place, giving a 100 % similarity to the reference sequence (GenBank: NM_014590). For cloning purposes, PCR reaction was repeated at identical conditions as described above, but less error-prone Pfu polymerase was used instead of less accurate Taq polymerase (Thermo Scientific, Lithuania). Furthermore, Pfu polymerase generated blunt-ended amplicons that were essential for the subsequent blunt-end cloning procedure.

In the next step, site-directed mutagenesis was made in order to introduce three point mutations into appropriate positions in the ERWVE1-specific PCR product making this region identical with that of MRSV (Fig. 1). To introduce point mutations, we utilised a megaprimer mutagenesis technique as described previously by Brons-Poulsen et al. [27]. The megaprimer method consists of two steps of polymerase chain reaction, where the first round of PCR generates a fragment containing the desired mutation. For the first PCR, Pfu polymerase was used (Thermo Scientific, Lithuania) and the reaction mixture consisted of 1xPfu Buffer, 2 mM of MgSO_4_, 200 nM of each deoxyribonucleotide, 1 U of Pfu polymerase and of 25 pmol of each syncytin_mut_F and a mutagenic_MRSV_R primer (Table 1). The reaction was performed in total volume of 25 μl under the following thermal conditions: 95 °C/5 min, 35 repeats of (95 °C/30 s., 58 °C/30 s, 72 °C/40 s.) and 72 °C/10 min. After amplification, the reaction product (megaprimer) was resolved by agarose gel electrophoresis and purified by means of silica-based spin columns. Obtained megaprimer’s amplimers were semi-quantified using Ethidium bromide-containing agarose plates and lambda DNA as a quantitative standard of known DNA copy number (Thermo Scientific, Lithuania) [28]. Reaction components of the second polymerase chain reaction were identical as in the first PCR, but one pmol of syncytin_mut_R primer and one microlitre of megaprimer solution were used as primers for Pfu polymerase (i.e. both megaprimer and reverse primer were in similar concentrations in the reaction mixture). Thermal settings of second PCR were adopted from the work of Brons-Poulsen et al.: 94 °C/2 min, 23 repeats of (94 °C/45 s, 58 °C/2 min, 72 °C/40 s) and 72 °C/10 min. Again, reaction products were resolved in agarose gel and a specific band of appropriate molecular weight (i.e. of 270 bp) was excised from the gel, purified as described previously and used directly for cloning parallel with ERVWE1 fragment.

### Construction and Preparation of MSRV and ERVWE1 Plasmids Employed as a Reference and for the Optimization of PNA-Mediated PCR Clamping

Both ERVWE1–derived and mutated MSRV-like PCR products were cloned into pSC-B-amp/kan plasmid using StrataClone Ultra Blunt PCR Cloning Kit according to the manufacturer’s guide (Agilent Technologies, USA). Mini preps were prepared in 3.5 ml of Luria Broth medium and were purified by NucleoSpin Plasmid kit (Macherey–Nagel, Germany). Obtained plasmids were given an initial examination to confirm the presence of the insert (regardless of their orientation) by PCR with a plasmid-specific universal T3/T7 primer pair. Finally, pSC-B-MSRV and pSC-B-ERVWE1 positive clones were sequenced in both directions by means of dideoxy-sequencing with the use of insert-specific primers, i.e. Syncytin_out_F and Syncytin_out_R (Genomed SA, Warsaw, Poland). The mini-prep extracts were quantitatively measured using SYBR Green 1 fluorescent dye (Life Technologies, Warsaw, Poland) against the known amounts of lambda DNA as a reference (Thermo Fermentas, Vilnius, Lithuania) in a fluorometer (Fluoroscan microplate reader, Labsystems, Finland). The excitation wavelength of the dye was 495 nm, and emission was filtered using a 520-nm barrier filter. Plasmid DNA copy number was then calculated using dsDNA copy number calculator (http://cels.uri.edu/gsc/cndna.html).

### The Synthesis of Complementary to ERVWE1 Peptide Nucleic Acids

All reagents and solvents were of analytical or HPLC grade. Solutions were freshly prepared with distilled, deionised water using a Milli-Q Millipore system (Millipore Corporation, Billerica, Massachusetts, USA) and filtered with a 0.22-μm syringe filter before use. Fmoc-XAL-PEG-PS resin for the PNA synthesis was obtained from Merck KGaA (Darmstadt, Germany). Fmoc/Bhoc-protected PNA monomers were purchased from Panagene (Billingham Cleveland, United Kingdom). DNA oligonucleotides were obtained from Future Synthesis (Poznań, Poland). The other reagents and solvents were obtained from Sigma-Aldrich Co. (Poznań, Poland).

PNA sequence: TACCAGTTTGGGTGA-amide complementary to ERVWE1 was synthesised using a Labortec AG SP-650 Peptide Synthesizer on a 3.8 µmol scale using a 2.5-fold molar excess of the Fmoc/Bhoc-protected monomers and Fmoc-XAL-PEG-PS resin (amine groups loading of 0.19 mmol/g) [29]. Monomers were activated with the use of a HATU/NMM/2,6-lutidine (0.7:1:1.5) mixture and coupled for 30 min as active derivatives. Fmoc deprotection was performed with the use of 20 % piperidine in DMF (2 × 2 min). Deprotection of the Bhoc group and cleavage of PNA from resin was carried out with the use of a TFA/m-cresol (95:5) mixture for 30 min.

The crude oligomer obtained was lyophilised and purified by semi-preparative reverse phase, high performance liquid chromatography RP-HPLC using a Knauer system with a Kromasil C8 column (20 × 250 mm, 5 µm particle size) (Sigma-Aldrich Co., Poznań, Poland). The mobile phase consisted of 0.08 % TFA in acetonitrile (solvent A) and 0.1 % TFA in water (solvent B). The gradient profile for the mobile phase was as 0–30 % of solvent A held for 90 min. The column was maintained at ambient temperature. The flow rate was 4.5 ml/min, and the eluate was monitored with a UV detector at 254 nm. Fractions, which had purity greater than 98 % were collected and lyophilised.

All analytical RP-HPLC analyses were performed on a Phenomenex Kinetex XB-C18 column (Phenomenex- Shim-Pol, Izabelin, Poland) (4.6 × 150 mm, 5 µm particle size) using a Beckman Gold System. The mobile phase consisted of 0.08 % TFA in acetonitrile (solvent A) and 0.1 % TFA in water (solvent B). The gradient profile for the mobile phase was as 0–30 % of solvent A held for 30 min. The column was maintained at ambient temperature. The flow rate was 1 ml/min, and the eluate was monitored with a UV detector at 254 nm.

Finally, PNA was characterised by MALDI-TOF mass spectrometry (Bruker, BIFLEX III), which confirmed the identity of the product synthesised.

### The Capillary Electrophoresis of PNA and Its DNA Homologues

The specificity of PNA hybridisation to the complementary ERVWE1 ssDNA fragment and not complementary MRSV ssDNA fragment was analyzed and confirmed by capillary electrophoresis [30]. Separations were performed using a P/ACE System MDQ (Beckman Instruments, Fullerton, CA, USA) controlled by Karat software. A coated eCAP DNA capillary (Beckman Coulter, USA) of 39.5 (29.5 to detector) cm × 100 μm thermostated at 35 °C was used. Analyses were performed at 7.8 kV using a background electrolyte (BGE) of dsDNA 1000 Gel Buffer (Beckman Coulter, USA) with reverse electrodes polarisation (anode at the detector end). Samples were introduced to the capillary at its anodic end by electrokinetic injection at 5 kV for 5 s. The capillary was rinsed with a new portion of a separation buffer between runs for 5 min. PNA/DNA hybrids were monitored with a UV detector at 254 nm. All experiments were performed in triplicate.

### Polymerase Chain Reaction and Its Inhibition by ERWVE1 (syncytin-1)-Specific PNA

To optimise the proper reaction conditions for an efficient, PNA-mediated PCR clamping, 1 × 10^5^ copies of pSC-B-ERVWE1 and of pSC-B-MRSV plasmids were subjected, in parallel, as the template into polymerase chain reactions. Reaction mixtures contained 1× Qiagen PCR Master Mix, 200 nM of Syncytin_out_F and 200nM of an appropriate reverse primer (sync1_REV/-2 or sync1_REV/-1 or sync1_REV/0, respectively, see Table 1 for details).

In our previous experiment, we found that the addition of PNA annealing step to a standard 3-step PCR in PNA-mediated, end-point PCR clamping assay cycle gave excellent results, much better as those in 3-step PCR [31]. After optimization, the reaction’s thermal conditions were finally set as follows: 95 °C/5 min, 26 repeats of (95 °C/30 s, 63 °C/50 s,58 °C/30 s, 72 °C/40 s) and 72 °C/10 min. In the next step, the smallest but still effective amount of ERWVE1- complementary PNA was estimated. Two types of peptide nucleic acid molecules were taken into consideration that differ by only one monomer at the C′-end. Final concentrations in the range of 0.05–5 μM PNA in PCR mixture were examined. Control reaction was supplemented with an appropriate amount of 5 % dimethyl sulfoxide (DMSO) instead of PNA. All reactions were performed in duplicate and analysed in 1.5 % agarose gel stained with ethidium bromide (0.5 μg/ml).

### Real-Time PCR

Real-time PCR with the hydrolysis probe and/or with PNA was performed using a Roche real-time PCR system; model LightCycler 480 (Roche Diagnostics Polska, Warsaw, Poland). A hydrolysis probe, complementary to MSRV but not to ERVWE-1 gene fragment was designed using EPRIMER3 software available from the EMBOSS package [22]. The target sequence for the probe hybridisation was overlapped with that for PNAs.

The hydrolysis probe used in the assay possessed a reporter dye (6-carboxyfluorescein, FAM) and a non-fluorescent quencher (Black Hole Quencher^®^-1, BHQ-1) at the 5′ and 3′ ends, respectively. The reporter dye’s emission is quenched when the probe remains intact. Only if the probe hybridised with its complementary target (i.e. MSRV *env* gene), the fluorescence of a reporter dye becomes detectable due to 5′ → 3′ exonuclease activity of Taq polymerase. In the case of MSRV, the hybridisation process of the probe is not impeded by PNA because of its non-complementarity to MSRV sequence (Fig. 1). On the contrary, the synthesis on the ERVWE1 locus/template cannot take place for two reasons: first, because the fluorescent probe is complementary to the MSRV but not to the ERVWE1; second, because PNA molecules hybridise with high affinity to the ERVWE1 template and thus prevent hybridisation of the hydrolysis probe. Thermal conditions for the quantitative analysis with the use of hydrolysis probe differ from that used in the end-point PCR. This results from the fact that the probe is hydrolysed during the primers’ annealing step. Finally, the conditions of the PCR were as follows: 10 min at 95 °C for the initial denaturation, followed by 50 cycles at 95 °C for 30 s for denaturation and 61 °C for 1 min for primer extension (with signal acquisition at this step). Reaction mixtures consisted of 1× Qiagen PCR Master Mix (containing the 1.5 mM of MgCl_2_), 2.5 mM MgCl_2_, 200 nM of each Syncytin_out_F forward- and sync1_REV/-1 reverse primer, 400nM of MSRV-complementary hydrolysis probe, 300nM of PNA (or an appropriate volume of 5 % DMSO).

### Preparation of Standard Curve for the Measurement of MSRV Using a Hydrolysis Probe

**Fig. 2:**
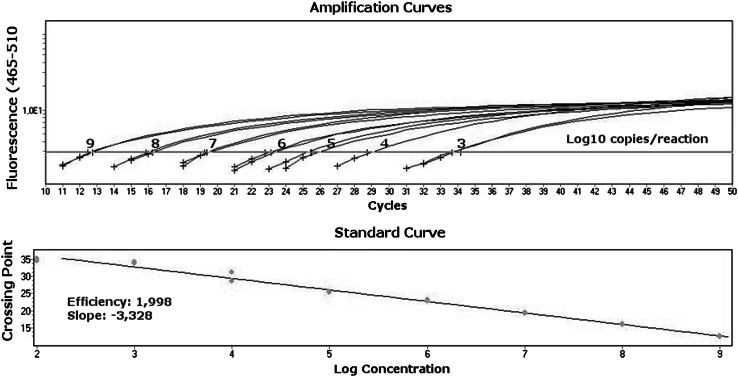
Standard curve prepared for the quantitation of MSRV. Plasmids contained MSRV env gene fragment (pSC-B-MSRV) that had been quantitatively adjusted were used for sevenfold serial dilutions from 1 × 10 e^9^ copies/reaction to 1 × 10 e^3^ copies/reaction; from these, the standard curves were generated. A strong inverse correlation between the logarithmic concentration of samples and CT values was obtained, and linearity was confirmed within a whole range of standard concentrations. The efficiency of the reaction reaches a value of 1.998

PSC-B-MSRV plasmids that had been quantitatively adjusted using an fluorometer (Fluoroscan microplate reader, Labsystems, Finland) beforehand were used for tenfold serial dilutions from 1 × 10 e^9^ copies/reaction to 1 × 10 e^2^ copies/reaction; from these, the standard curves were generated (Fig. 2).

## Results

### PNA-Mediated PCR Clamping of ERWVE1 Plasmid DNA Template

**Fig. 3:**
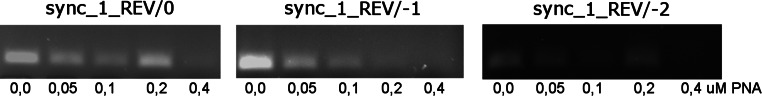
The optimization of reaction conditions of PNA-mediated PCR clamping. The specific inhibition of ERVWE1 by an assay relies on exact reciprocal probe-to-primer localization and on the optimal concentration of the PNA. The best results were obtained if a PNA probe shared only one base with the adjacent reverse primer (sync_1_REV/-1)

In our previous experiments, we have documented that the amplification of ERVWE1 gene fragment by means of polymerase chain reaction (PCR) can be specifically inhibited by complementary peptide nucleic acids (PNA) while no inhibition occurred when MSRV clones were used as the template. Furthermore, we also found that slight differences in PNA design or in primers localization altered drastically the efficiency of PCR blocking. A PNA probe, 14-monomers in length that hybridise at the position 1370–1383 in respect to the reference gene (GenBank: NM_014590) efficiently clamped the amplification of ERVWE1 at the concentration of at least 4 μM while we noted that its longer analogue (15-monomers, hybridised at positions 1369–1383) worked effectively even at tenfold lower concentration of 400nM or less (Fig. 3).

In theory, PCR clamping operates by three possible mechanisms of action, i.e.: (1) by the competition for a common target site between a PNA and one of the PCR primers; (2) by the interference of primer elongation and/or (3) by the product elongation arrest. According to our observations, primer elongation stoppage was the only efficient way to inhibit the reaction. Neither the competition between PNA and one of the PCR primer for a common target nor the product elongation arrest was efficient to inhibit the reaction even at the final PNA concentration of as much as 10 μM. Even in the case of an inhibition caused by primer elongation stoppage, the efficiency of the clamping was strongly influenced by the exact reciprocal primer-to-PNA localization (Figs. 1, 3).

### Real-Time PCR of MSRV Template

Standard curve for the quantitative measurement of MSRV template in the sample was prepared using serial dilutions of the pSC-B-MSRV plasmid stock from 1 × 10 e^9^ copies/reaction to 1 × 10 e^2^ copies/reaction. A strong inverse correlation between the logarithmic concentration of samples and Ct value was obtained, and linearity was confirmed within a range from 1 × 10 e^3^ to 1 × 10 e^9^ copies/reaction (Efficiency: 1.998; Slope: −3.328, Fig. 2). All standard concentrations were run in duplicates.

### Measurement of a Target MSRV in the Presence of ERVWE1 Sequence

**Fig. 4:**
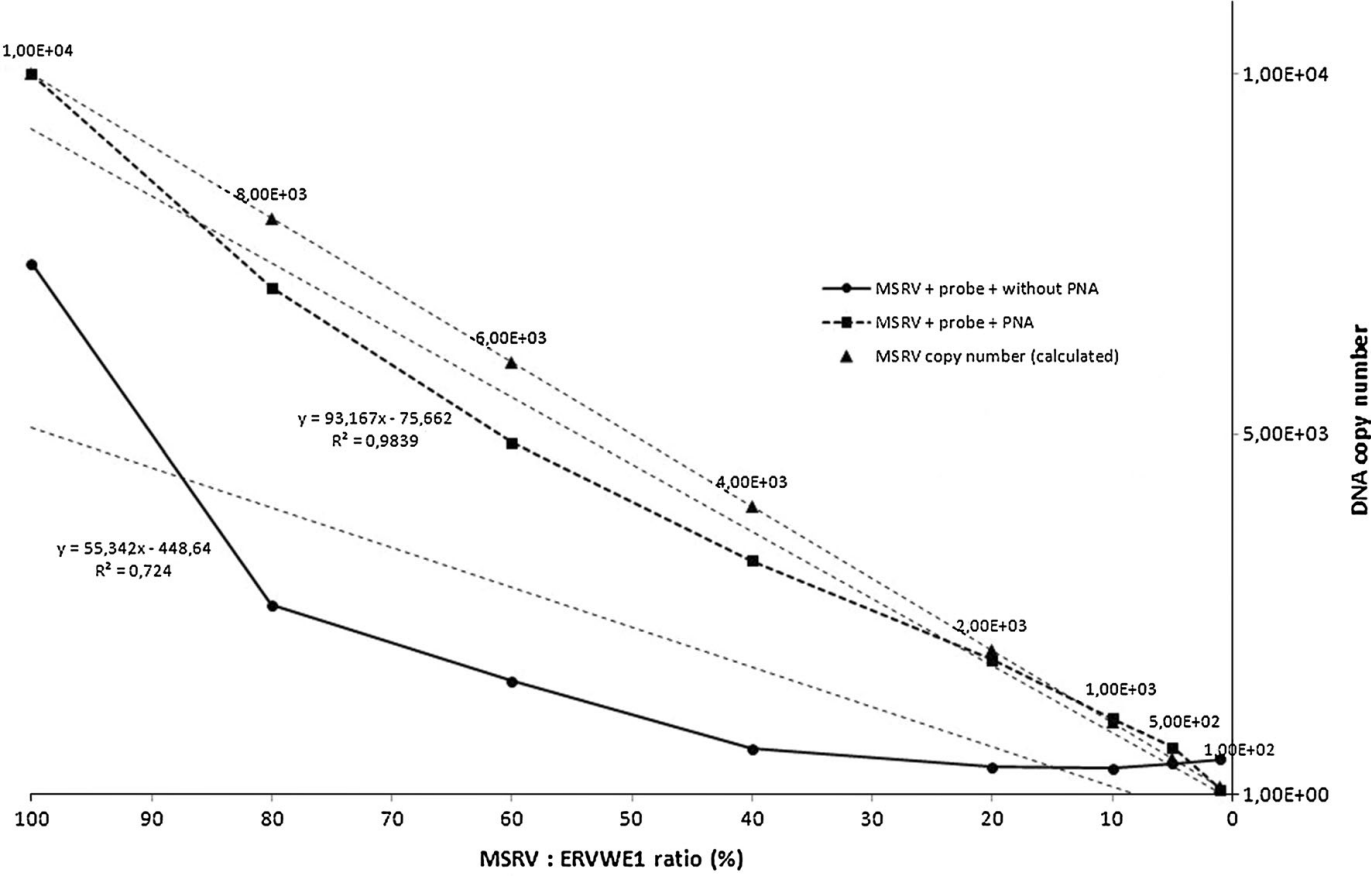
The measurement of a target MSRV sequence in mixed templates. Serially diluted standard templates were amplified in the presence or absence of ERVWE1-specific PNA during probe-based real-time PCR to measure the MSRV expression level. An amount of 1 × 10 e^4^ copies/reaction of plasmid DNA containing MSRV fragment (pSC-B-MSRV) was used as template and 0.4 μM of ERVWE1-specific PNA was added into the reaction mixture in this assay. The pSC-B-MSRV was then serially diluted with plasmid DNA that contained ERVWE1 sequence (pSC-B-ERVWE1). The proportions of pSC-B-MRSV were adjusted to 100, 80, 60, 40, 20, 10, 5 and 1 % (pSC-B-MSRV:pSC-B-ERVWE1 ratio). Control samples that did not contain PNA were analysed parallelly. In PNA-containing samples, a linearity of the plot was maintained in the whole range of MSRV concentration values but if no PNA was added into the reaction, a correct quantitation of MSRV was possible only if an amount of MSRV DNA was equal to 100 % in respect to ERVWE1 but not at the lower MSRV-to-ERVWE1 values when the measurement was not linear. The accuracy of the method was evaluated according to the calculated plasmid copy number (depicted as *triangles* at the plot). All samples were run in duplicates

The accuracy of quantification of MSRV DNA was determined in a mixed MSRV/ERVWE1 template by real-time PCR with a MRSV-specific probe with or without PNA. Briefly, 1 × 10 e^4^ molecules of the pSC-B-ERVWE1 plasmid DNA were serially diluted with the pSC-B-MSRV plasmid DNA of the same stock concentration. Proportions of pSC-B-MRSV were adjusted to 100, 80, 60, 40, 20, 10 and 1 % (pSC-B-MSRV to pSC-B-ERVWE1 ratio). If PNA was not present in the reaction, the target MSRV sequence was correctly measured only at the highest concentration of the MSRV compared to ERVWE1 and an estimated molecule number was similar to that theoretically calculated only in these experimental conditions. Measurements were underestimated when the concentration of MSRV template was lower (Fig. 4). On the contrary, the addition of ERVWE1-specific PNA probe to the reaction improved greatly the accuracy of the method and allowed to estimate the MSRV copy number even if this template was in minority in respect to ERVWE1. Additionally, a linearity of the assay was maintained.

### Evaluation of the Method Using Biological Samples

In the next step we examined the level of MSRV mRNA copy number in human cells using both hydrolysis probe and PNA. As a target for the assay we chose those cell lines, in which specific blocking of ERVWE1 amplification may be crucial to monitor the discrete expression changes of MRSV irrespective of the ERVWE1 transcription. It was documented that various human tissues and cell lines exhibit an overall expression of HERV-W *env* elements but their highest mRNA level was observed in placenta, glial cells and in testes [32]. Regardless the cell type, an altered expression of human endogenous retroviral W family was observed in numerous pathological conditions, such as neuroinflammation [33], burn-caused injury [34] or exogenous viral infection [35, 36].

Based on our previous observations, we checked if the expression of MSRV was detectable and could be measured quantitatively in normal human astrocytes (NHA) and in human umbilical vein endothelial cells (HUVECs) cultured in vitro. We also examined whether the MSRV expression level changed after the lipopolysaccharide (LPS) - induced activation of human monocytes in vitro.

**Fig. 5:**
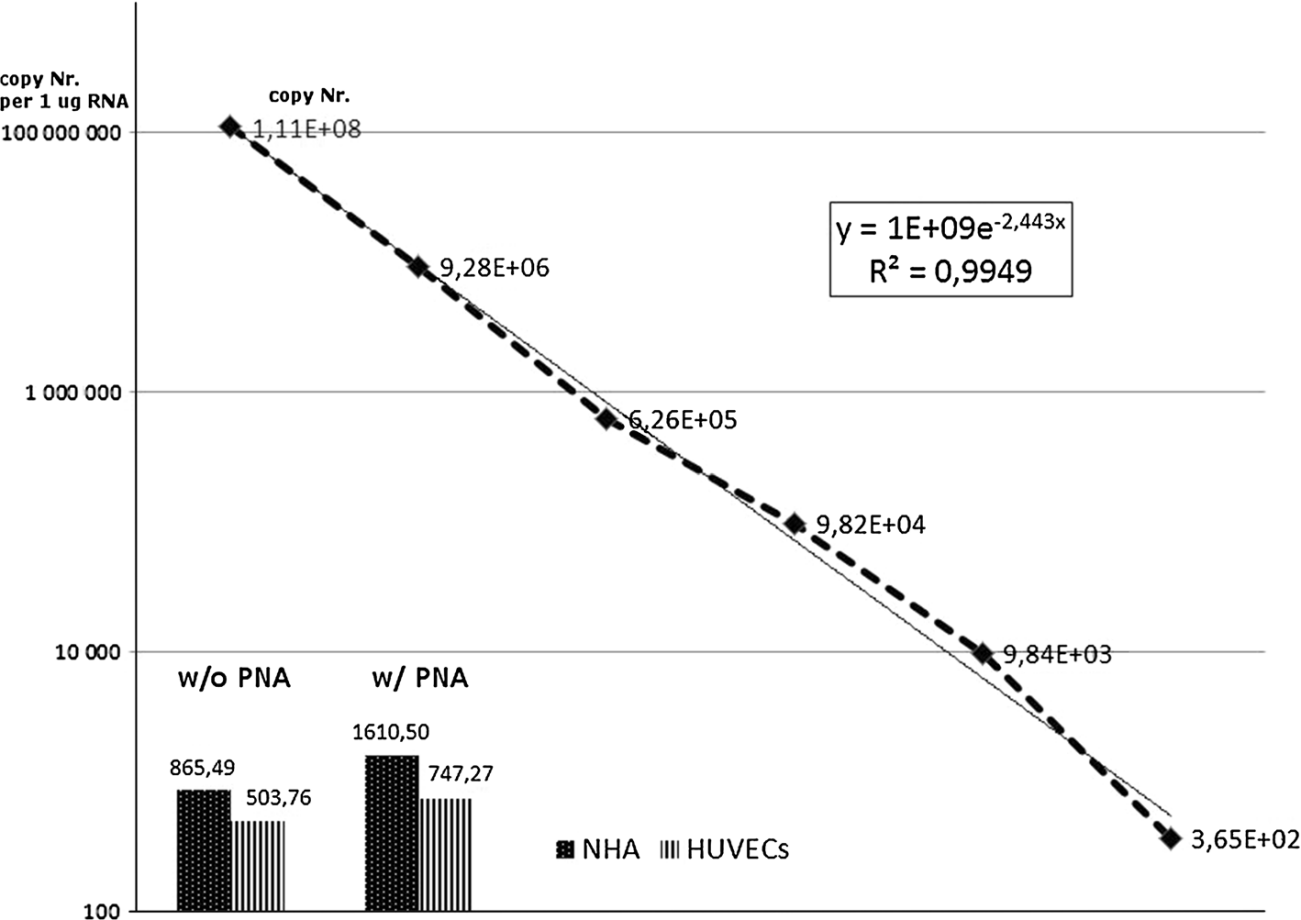
The quantitative analysis of MSRV mRNA in cell lines. An amount of MSRV mRNA was estimated in normal human astrocytes (NHA) and in human umbilical vein endothelial cells (HUVECs) in in vitro cultures. The addition of PNA into the reaction increased the calculated MSRV copy number in both NHA and HUVECs when compared with the quantitative analysis without the use of PNA. These results indicate that an inhibitory effect caused by ERVWE1 present in the sample may be eliminated by the use of ERVWE1-specific PNA and that ERVWE1 is more abundantly expressed in NHA than in HUVECs (1.86- and 0.67-fold increase in calculated mRNA copies number after addition of PNA, respectively). For the quantitative analysis of MSRV in cells, an individual, in-run standard curve was generated in the range from 1 × 10 e^8^ copies/reaction to 1 × 10 e^2^ copies/reaction (*dotted line* with data labels)

We confirmed that the expression of MSRV was present in investigated cell types albeit its level was relatively low in NHA and HUVECs 1.61 × 10 e^3^ ± 2.88 × 10 e^2^ and 7.47 × 10 e^2^ ± 3.4 × 10 e^1^ mRNA copies number/μg RNA, respectively. For beta actin gene expression, the results were as follow: 1.29 × 10 e^5^ ± 1.54 × 10 e^4^ and 2.52 × 10 e^4^ ± 1.4 × 10 e^3^ mRNA copies per one microgram of total RNA, respectively. As we expected, the measurement values of MSRV mRNA copy number change if ERVWE1- specific PNA was added into the QPCR reaction mixture simultaneously with MSRV-specific fluorescent probe. In the control reactions where no PNA was added but only probe was present, the obtained results were lower in comparison with that after the addition of PNA (NHA: 8.65 × 10 e^2^ ± 1.6 × 10 e^1^ mRNA copy number/μg RNA; HUVECs: 5.03 × 10 e^2^ ± 2.6 × 10 e^1^ mRNA copy number/μg RNA) (Fig. 5) indicating that the possible reaction inhibition by an abundant ERVWE1 expression was done.

**Fig. 6:**
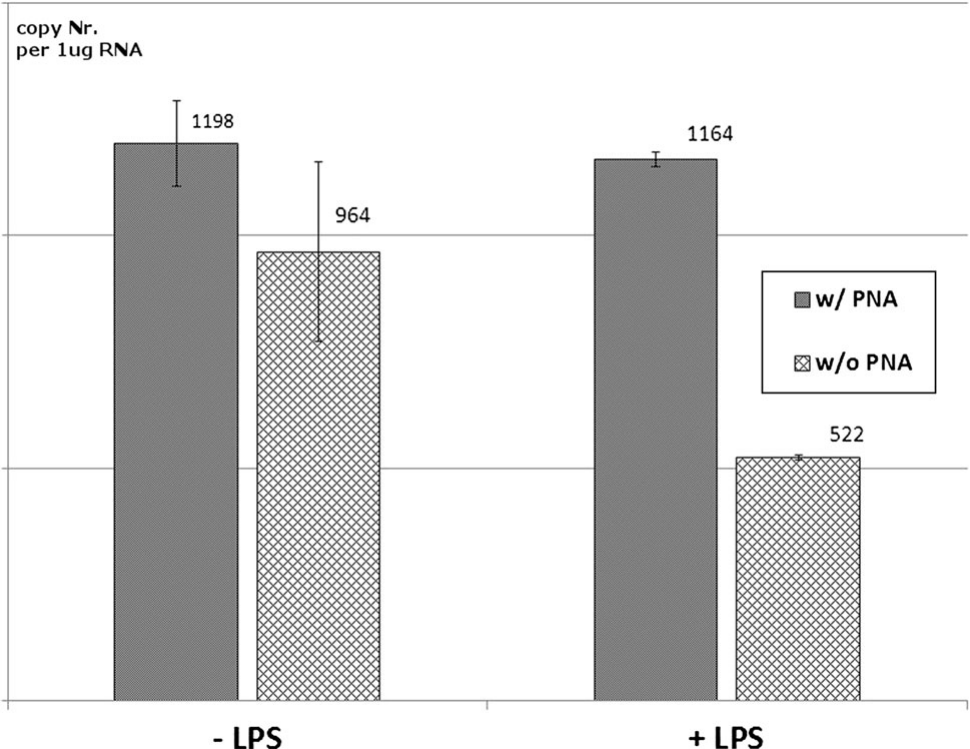
The use of PNA-mediated, Real-Time PCR for the quantitation of MSRV mRNA copy number in human monocytes after (LPS)-induced activation. The calculated MSRV mRNA copy number did not change in the QPCR quantitation method that utilised PNA regardless the stimulation by LPS or not. On the contrary, in standard probe-based QPCR without PNA, a strong decrease of MSRV expression level was noted, indicating that LPS may influence the ratio between MSRV and HERV-W *env* expression in human monocytes especially by the diminishing of non-MSRV-derived HERV-W copy number

In human monocytes, the stimulation by lipopolysaccharide (LPS) did not alter the expression of MSRV because no differences in HERV-W expression were observed after addition of PNA into the reaction (1.198 × 10 e^3^ ± 1.83 × 10 e^2^ vs. 1.16 × 10 e^3^ ± 3.2 × 10 e^1^ mRNA copy number/μg RNA). Interestingly, an inhibitory effect of LPS on the expression of HERV-W was observed in samples without PNA against ERVWE1 (9.64 × 10 e^3^ ± 1.93 × 10 e^2^; vs. 5.22 × 10 e^3^ ± 6.0 × 10 e^0^ mRNA copy number), indicating that LPS may influence the ratio between MSRV and HERV-W *env* expression in human monocytes, especially by the diminishing of non-MSRV-derived HERV-W copy number (Fig. 6).

## Discussion

The endogenous retrovirus HERV-W family is broadly represented in human genome; its sequences have been detected on almost all chromosomes. However, currently, only two loci are thought to play a specific role in both human physiology and pathology. All the explored HERV-W sequences are closely related and are difficult to distinguish [10].

Phylogenetic study of HERV-W sequences derived from brain and peripheral blood mononuclear cells of multiple sclerosis (MS) patients provided by Mameli et al. showed that envelope gene intracellular domain of MSRV and ERVWE1 (syncytin-1) RNA share >89 % similarity while whole envelope gene showed >93 % similarity in both MSRV and ERVWE1.

The homology between MSRV *env* and syncytin-1 gave rise to speculations regarding their possible physiologic and/or pathogenic effects. A major difference is that only MSRV has been detected as an extracellular virus (visualised by electron microscopy, with a polyA (+) RNA containing terminal repeats, gag, pol and *env* sequences) while the syncytin-1 protein has been found only intracellularly or on the plasma membrane [37].

As a result of high similarity at the protein level, no specific antibody for unique HERV-W family members has been identified so far and it is not possible to discriminate between MSRV *env* and syncytin-1 on the protein level. Thus, any results based on immunostaining or Western blot could reflect either MSRV *env* or syncytin-1 expression, or both.

Another approach to distinguish between the expression of MSRV *env* and syncytin-1 in research assays is to utilise PCR, reverse transcriptase (RT) PCR and/or real-time PCR assays that could selectively amplify either MSRV or syncytin-1 mRNA sequences. Reliability of the assays with respect to specificity, sensitivity and inter- and intra-assay variations has been published previously [11]. With respect to ERVWE1, MSRV *env* sequences harbour a 12-nucleotide insertion in the transmembrane moiety. Based on this insertion, discriminatory real-time PCR assay was developed that can selectively amplify either MSRV *env* or ERVWE1. The selectivity of the method was achieved by the use of allele-specific primer pairs and appropriate probes, TaqMan-type for MSRV and ERVWE1 and locked nucleic acid (LNA) probe for the generic HERV-W *env* [10].

A similar approach was demonstrated by Antony et al., where authors employed a real-time PCR approach using MRSV or syncytin-1-selective oligonucleotide primers and fluorescent, intercalating SYBR Green I dye which allowed the quantitative analysis of gene expression in brain tissue samples, peripheral blood leukocytes (PBLs) and monocyte-derived macrophages (MDM) from multiple sclerosis (MS) and non-MS patients as well as in an astrocytic U373 cell line. Analyses provided by Antony et al. revealed that ERVWE1 *env*-encoding DNA and RNA exhibited statistically significant increased detection and expression in the brains of MS patients in comparison with control. Moreover, ERVWE1 *env* transcripts were inducible in glial cells as well. Conversely, such changes in MSRV expression were not reported in that work [13].

Due to an error-prone nature of Taq polymerase, a reverse transcriptase-quantitative polymerase chain reaction based on intercalating dyes is not recommended for the detection and quantification of mutant nucleic acids, especially when slight differences among investigated sequences are expected (such as in the case of single-nucleotide mutations, SNPs). Any suboptimal reaction conditions (temperature fluctuations at the primers annealing, the overload of an enzyme co-factor MgCl_2_ etc.) may result in possible primers-to-template mishybridisation and, subsequently, to the synthesis of unspecific amplimers [38]. Therefore, in the allele-specific amplification PCR (ASO) where allele- specific primers are used, many of 3′ mismatches between a primer and its template do not efficiently impair the PCR reaction of the wild-type template and may giving false-positive signals [15].

In order to improve the specificity of the assay, the use of SYBR Green I has to be replaced by a sequence-specific fluorescent probe. Until now, SYBR Green 1 dye has been commonly used because it is simple to use, sequence-independent (there is no need to design any sequence-specific probe) and generates fluorescent signal, which theoretically is proportional to the number of double-stranded DNA molecules that are produced during PCR. This dye is suitable in most typical, quantitative, nucleic acid- based assays. On the contrary, if the reaction conditions are not optimal and/or if any double-stranded DNA molecules other than that from desired template arise in the reaction mixture, SYBR Green I gives inappropriate, false-positive results. Indeed, in respect to HERV-W research, Garson et al. in his Letter to the Editor of “AIDS Research and Human Retroviruses” journal ascertain some technical flaws in the real-time PCRs previously employed in the studies performed by Antony et al. [13, 39]. Thus, in our opinion, in MSRV analysis the best way to obtain much more accurate results is the use of PNA concomitantly with fluorescent probe and perform a direct quantification method instead of relative quantification based on intercalating dyes. In our assay, the addition of a sequence-specific fluorescently labelled oligonucleotide probe enhances greatly the selectivity of the reaction, as there are three independent factors that must occur simultaneously to give specific level of fluorescence.

Peptide nucleic acid (PNA) is an artificially synthesised polymer that has properties of both nucleic acids and proteins. Importantly, PNA binds to the complementary sequence of nucleic acid with much stronger binding capacity than DNA does, because of the lack of electrostatic repulsion.

Thus, in our assay, PNA oligomer is intended to bind specifically to the cDNA of the ERVWE1 locus and prevent them from being amplified by polymerase action. Our methodology allows the detection and measurement of discrete MSRV expression level due to MSRV-specific fluorescent oligonucleotide. What is more, any ERVWE1- derived templates are excluded because of their specific clamping by PNA. These factors improve greatly the analysis of MSRV quantity even if it is present in minority in respect to other similar molecules.

In our previously published data, we found that SYBR Green I-based Real-Time PCR and its specific clamping by ERVWE-1-specific PNA favour the phenomenon in which virtually all “non-ERVWE1-derived” amplimers could be synthesised. Because only two HERV-W members, namely MSRV and ERVWE1 comprise the majority of HERV-W expression products at all, the rest of amplimers that arose and that are quantified in course of the Real-Time PCR are, in theory, considered as being of MSRV origin. However, any possible expression from distinct HERV-W loci cannot be excluded. The principle of an assay changes if non-specific SYBR Green-I dye is replaced by a MSRV-specific oligonucleotide probe. Thus, the fluorescent signal source for the quantification of the results is derived exclusively from the amplimers synthesised on MSRV template due to the reciprocal, independent hybridisation of both primers and the probe. To sum up, PNA molecules play a role as “scavenger inhibitors” that alleviate the impact of substantial ERVWE-1 overload that could interfere with the reaction efficiency (see Fig. 4, dotted line vs. solid line).

## Conclusions

We developed a novel, real-time PCR technique that uses a syncytin-1 (ERVWE-1)-specific PNA oligomer and MSRV-specific fluorescent probe. This technique is intended for the discrete detection and measurement of MSRV (multiple sclerosis-associated retrovirus) expression level in biological samples regardless of syncytin-1 expression from ERVWE-1 locus.
